# Grape pomace as a novel functional ingredient: Mitigating ochratoxin A bioaccessibility and unraveling cytoprotective mechanisms *in vitro*

**DOI:** 10.1016/j.crfs.2024.100800

**Published:** 2024-06-25

**Authors:** Luciano Mangiapelo, Massimo Frangiamone, Pilar Vila-Donat, Denisia Paşca, Federica Ianni, Lina Cossignani, Lara Manyes

**Affiliations:** a Department of Pharmaceutical Sciences, Section of Food Science and Nutrition, University of Perugia, 06123, Perugia, Italy; b Department of Biomedical Sciences, University of Lausanne, Rue du Bugnon, 1005, Lausanne, Switzerland; c Laboratory of Food Chemistry and Toxicology, Facultat de Farmàcia i Ciències de l’Alimentació, Universitat de València, 46100, Burjassot, Spain; d Bromatology, Hygiene, Nutrition, Department 3 - Pharmacy, Faculty of Pharmacy, “Iuliu Hațieganu” University of Medicine and Pharmacy, Cluj-Napoca, Romania

**Keywords:** Mycotoxins, Jurkat T, Caco-2, Flow cytometry, Oxidative stress

## Abstract

Mycotoxins, secondary metabolites produced by molds, pose significant health risk through contamination of globally consumed cereals. Ochratoxin A (OTA), a prevalent mycotoxin in cereals, is associated with various health hazards, including immunotoxicity. This study explores the bioaccessibility of OTA in bread and its impact on the gastrointestinal barrier. A focus is placed on grape pomace (GP), a by-product of the wine industry, as a potential mitigator of OTA toxicity. Results demonstrate that GP reduces OTA bioaccessibility in the human gastrointestinal system from 94% to 81% at intestinal level, showing promise in limiting the absorption of the harmful toxin. Additionally, GP exhibits cytoprotective effects, enhancing cell viability and mitigating OTA-induced toxicity in both Caco-2 and Jurkat T cells. In view of the above, to understand the mechanisms by which OTA exhibits its toxic effects, flow cytometry was chosen as the main technique for the analysis of cell cycle, reactive oxygen species levels and mitochondrial parameters. Cytofluorimetric evaluation indicates GP's potential in limiting OTA-induced damage at cellular level. The study suggests that GP could serve as functional ingredient to reduce mycotoxin bioaccessibility and toxicity in cereal-based foods, offering a novel and promising approach to enhance food safety and protect public health. The finding highlights the potential of utilizing grape pomace in food formulations to mitigate mycotoxin contamination, providing a valuable contribution to the ongoing efforts to ensure the safety of globally consumed cereal products.

## Highlights

• Barley grains were contaminated with *A. steynii* to obtain OTA contaminated flour.
• Realistic scenario and OTA concentration for all exposures *in vitro.*
• OTA bioaccessibility was reduced 13% when 2% grape pomace was added in bread recipe.
• Grape pomace addition to OTA bread reduced cell death to control levels in Jurkat model.
• Grape pomace decreased ROS generation in Jurkat cells produced by OTA exposure.

## Graphical abstract

**Figure:**
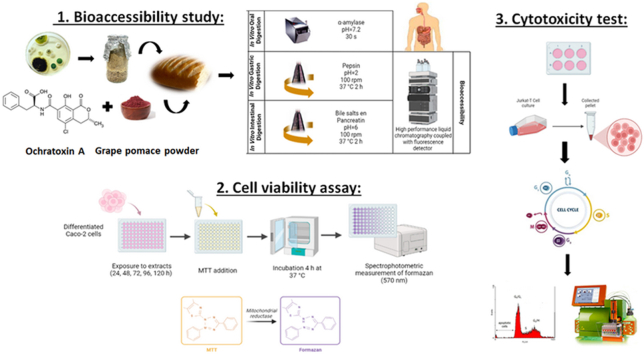

## 1 Introduction

Mycotoxins are toxic secondary metabolites produced by several species of molds, which can contaminate a wide range of food products, such as cereals, posing severe health risks to humans and animals. Cereals and byproducts are extensively consumed worldwide, making mycotoxin contamination a critical concern for the food industry. Various types of mycotoxins, including aflatoxins, ochratoxins, deoxynivalenol, zearalenone, fumonisins, and patulin, are commonly found in cereals (Manyes and Font, 2023).

Ochratoxin A (OTA), a phenylalanyl derivative primarily produced by different species of *Aspergillus* genera, is known for its teratogenic, embryotoxic, genotoxic, neurotoxic, and immunosuppressive effects in various *in vitro* and *in vivo* models (Cimbalo et al., 2020). OTA has been also classified as a possible human carcinogen (Group 2B) by the International Agency for Research on Cancer (IARC). Furthermore, OTA is one of the most prevalent mycotoxins found in cereals and cereal-based products such as bread with a level of 2.69 μg/kg. It is also found in coffee (3.08–3.34 μg/kg), cocoa (1.55–3.88 μg/kg), spices (38.8 μg/kg), dried fruits (5.60 μg/kg), and red wine (1.46 μg/kg) (Kumar et al., 2020; Dundray and Pacin, 2013; Iqbal et al., 2018; Freire et al., 2020). To address OTA contamination and safeguard public health, European regulatory agencies and organizations have established maximum limits for OTA levels in unprocessed cereals (5.0 μg/kg), cereal-based products (3.0 μg/kg), roasted coffee (3.0 μg/kg), cocoa powder (3.0 μg/kg), dried spices (15 μg/kg), wine (2.0 μg/kg), and dried fruit except raisins and figs (2.0 μg/kg) (European Commission, 2023).

One of the key factors in mycotoxins risk assessment is the bioaccessibility, which represents the fraction of metabolites released from the food matrix into the gastrointestinal tract (Trujillo-Rodriguez et al., 2020). According to studies in literature involving *in vitro* human digestion models, the bioaccessibility of OTA in wheat bread was 7.7% in the gastric phase and 88% in the intestinal phase (Escrivá et al., 2021). In processed cereal-based foods for children consumption, OTA intestinal bioaccessibility ranged from 95% to 105% (Rebellato et al., 2021). Using a semi-dynamic gastro-duodenal digestion system with a combined meal of liquid yogurt and corn cookies, OTA intestinal bioaccessibility was found to be 72% (Sobral et al., 2022). It has been also reported bioaccessibility values of 95–100% in cornmeal, peanut slurry, and buckwheat (Versantvoort et al., 2005; Nobre et al., 2022).

Once reached the intestine, OTA can also disrupt the integrity of the gastrointestinal barrier (Assunção et al., 2019). Numerous *in vitro* and *in vivo* studies have demonstrated that OTA-exposure has been associated with the impairment of gastrointestinal barrier, mainly reproduced using differentiated Caco-2 cells, via intracellular reactive oxygen species (ROS) generation, apoptosis, and cell cycle alterations (Frangiamone et al., 2022a). Moreover, OTA can alter the permeability of gut barrier and reach the bloodstream, in which it may promote clear alterations on immune cells homeostasis. For instance, Frangiamone et al. (2023) demonstrated that OTA cytotoxicity was correlated with metabolic reprogramming and glycolysis activation in Jurkat cells.

Considering the high prevalence of OTA in cereal products and its toxic effects for human health, the scientific community tried to identify new strategies to mitigate mycotoxin contamination, adsorption and toxicity. A novel avenue of investigation involves the exploration of adsorbent materials that show a high affinity for mycotoxins and can reduce their gastrointestinal bioaccessibility (Li et al., 2018). In this context, grape pomace (GP), the main by-product of wine industry, represents approximately 20% of grapes used for winemaking and a putative novel dietary strategy for fungal contamination (Bordiga et al., 2019; Tolve et al., 2021). Indeed, innovative uses of GP have been proposed in the formulation of nutritious foods with high dietary fiber and polyphenol contents, particularly as functional ingredient in cereal-based products (Rocchetti et al., 2021; Sant’Anna et al., 2014; Boff et al., 2022). However, the possible cytoprotective role of GP against mycotoxins as well as its beneficial effects in several biological processes such as cell cycle, apoptosis, oxidative stress, and mitochondrial homeostasis have been rarely investigated. For the study of OTA toxicity in biological processes, flow cytometry is an extremely valuable tool which provides an excellent interpretation of ROS production, cell proliferation and apoptosis. It represents a useful technique that allows a rapid analysis of multiple chemical and physical cell characteristics (Zhou et al., 2020; Bleichrodt and Read, 2019). For this reason, flow cytometry was selected to evaluate changes in biological processes after mycotoxin exposure.

In view of the above, the aim of this study was to investigate the application of GP as a functional ingredient to reduce OTA bioaccessibility in the human gastrointestinal system as well as to evaluate its protective role against OTA toxic effects on cellular viability, cell cycle, cell death, ROS production and mitochondrial mass *in vitro*.

## 2 Material and methods

### 2.1 Chemicals and reagents

Grape pomace deriving from Cabernet grapes variety was provided by a local farmer company in 2021, located in the province of Perugia (Italy). Barley grain (Biogrà) was produced by P.D.R. Sorribas S.A.U., Polinyà, (Valencia, Spain), strength wheat flour (Alteza) was obtained by Haricaman, S.L. Ctra (Toledo, Spain), fresh yeast (Levanova) was produced by Lesaffre Iberica S.A. (Valladolid, Spain), white sugar from Pfeifer & Langen GmbH & Co. (Colonia, Germany), fine sea salt was manufactured by Polasal S.A. (Alicante, Spain). Acetonitrile (ACN), Methanol (MeOH) for HPLC and Acetic acid were supplied by Fisher Scientific (Madrid, Spain). Deionized water was generated by a Milli-Q water purification system (Millipore, Bedford, MA, USA). OTA standard solution, potassium chloride (KCL), 3-(4,5-dimethylthiazol)-2,5-diphenyltetrazolium (MTT), potassium thiocyanate (KSCN), sodium dihydrogen phosphate (NaH_2_PO_4_), sodium sulfate (Na_2_SO_4_), sodium chloride (NaCl), sodium hydrogen carbonate (NaHCO_3_), urea (CO(NH_2_)_2_), α-amylase (930 U/mg; A3403), hydrochloric acid (HCl), sodium hydroxide (NaOH), pepsin A (674 U/mg; P7000), pancreatin (762 U/mg; P1750), 2′,7′-dichlorodihydrofluorescein diacetate (H_2_DCFDA) ROS detection kit and bile salts (B8631) were purchased from Sigma-Aldrich (St. Louis MO, USA). DMSO, MitoTracker green M7514 kit (Invitrogen), MitoSOX for mitochondrial ROS generation kit and DMEM were purchased from Thermo Fischer Scientific (Waltham, Massachusetts, USA). Phosphate buffer saline (PBS) was purchased from Sigma Chemical Co. (St. Louis, MO, USA). Roswell Park Memorial Institute (RPMI) culture medium by Biowest (Nuaillé, France), Annexin V-FITC kit was purchased from Miltenyi Biotec (Bergish Gladbach, Germany), Cycletest™ Plus DNA Reagent Kit for the cell cycle assay by flow cytometry was purchased from BD Biosciences (San Diego, CA).

### 2.2 Barley flour contamination and OTA production

Contaminated barley flour was obtained using *A. steynii 20510* obtained from the Spanish Type Culture Collection (CECT). Mycelium and spore suspension of fungal strain were added to 1 L glass jars containing 400 g of barley grain. Glass jars were kept a room temperature and 5 mL of Milli-Q water were added every two or three days to maintain a constant level of humidity. To keep track of OTA production by the fungus, mycotoxin extraction from flour was carried out once a week and the extract evaluated by using high performance liquid chromatography coupled with fluorescence detector (HPLC-FLD).

### 2.3 Bread production

Lyophilized GP was combined with and without contaminated barley flour to produce four breads: Control (C), 2% GP, OTA, and OTA-GP. Breads were made by combining all the components shown in Table 1. A SilverCrest Bread Maker SBB 850 A1 (Kompernass GMBH, Bochum, Germany) was used to homogenize the doughs (each weighing approximately 100 g). Doughs were then introduced into molds, covered with a damp cloth, and fermented at room temperature for 1 h. After the fermentation process, doughs were placed in a Memmert ULE 500 muffle furnace (Madrid, Spain) and baked for 150 min at 120 °C.

**Table 1:** Recipes of Control (C), grape pomace (GP), OTA and OTA + grape pomace (OTA-GP) breads.

Ingredient (g)	C	GP	OTA	OTA-GP
Wheat flour	63.5	61.4	56.5	54.4
Contaminated barley flour	0	0	7	7
Water	33	33	33	33
Salt	1.3	1.3	1.3	1.3
Sugar	2	2	2	2
Fresh yeast	4	4	4	4
GP	0	2.1	0	2.1
Total quantity	**103.8**	**103.8**	**103.8**	**103.8**

### 2.4 *In vitro* static digestion model

To measure OTA bioaccessibility, an *in vitro* digestion model was used to replicate the whole human digestive process described in Escrivá et al. (2021). This process consists of three digestion phases: oral, gastric, and intestinal. Briefly, 10 g of grinded bread were combined with 6 mL of synthetic saliva and 84 mL of Milli-Q water (37 °C) in sterilized Stomacher® bags (IUL, Barcelona, Spain) for 30 s, simulating the mastication process. Saliva solution was obtained by mixing 1 mL of KCl (89.6 g/L), 1 mL of KSCN (20 g/L), 1 mL of NaH_2_PO_4_ (88.8 g/L), 1 mL of Na_2_SO_4_ (57 g/L), 0.17 mL of NaCl (175.3 g/L), 2 mL of NaHCO_3_ (84.7 g/L), 0.8 mL of urea (20 g/L), 29 mg of α-amylase and 2.5 mg of mucin and adjusted to pH 6.8 ± 0.2. Gastric phase was made by adding pepsin solution (1 g of pepsin in 25 mL of HCl 0.1 N) to the saliva after adjusting the pH to 2 ± 0.2. Gastric solution was added to Erlenmeyer flask containing oral digest and incubated in continuous stirring at 100 rpm for 2 h at 37 °C. After incubation, an aliquot of gastric digest was conserved at −20 °C for further analysis. Pancreatic digestion was simulated by adjusting the pH of the gastric phase to 6.5 ± 0.2 before adding bile salts and pancreatic solution (0.1 g of pancreatin and 0.625 g of bile salts were added to 25 mL of NaHCO_3_ 0.1 N). The content was moved to a second Erlenmeyer flask and kept in continuous stirring for 2 h at 37 °C. After the incubation, samples were stored at −20 °C. For each bread typology, the entire procedure was carried out three times (n = 3).

### 2.5 HPLC-FLD quantitative analysis

The quantification of OTA was made on an Agilent 1100 quaternary pump series coupled with an Agilent 1200 FLD detector, supplied with a vacuum degasser and an automatic sampler (Agilent Technologies, Santa Clara, CA, USA). A Kinetex EVO C18 column (150 × 4.6 mm, 5 μm particle size, 100 Å pore size) (Phenomenex, Palo Alto, CA, USA) was used as stationary phase. The column was conditioned for 20 min before use with the selected mobile phase and kept at 40 °C for the entire analysis. An isocratic mode of elution based on ACN/H_2_O/CH_3_COOH (55/43/2 v/v) as mobile phase solution was adopted with a flow rate of 0.8 mL/min. The injection volume was 20 μL for flour and breads extracts and 40 μL for gastric and intestinal extracts. The excitation and emission wavelengths were set to 330 and 460 nm, respectively. The good linearity is expressed by the high R^2^ values (>0.991) obtained for all the matrix-matched calibration curves.

### 2.6 Bread and contaminated barley flour OTA extraction

OTA extraction was carried out by adding an extraction mixture consisting of MeOH/H_2_O (80:20 v/v) to 10 g of bread or 5 g of contaminated barley flour. Samples were then ground with an Ultraturrax (T 18 digital ULTRA-TURRAX®, Staufen, Germany) for 3 min and centrifuged for 5 min at 3849 g (Centrifuge 5810R, Eppendorf, Germany). For HPLC-FLD analysis, the supernatant was filtered with a 0.22 μm syringe filter (Phenomenex, Madrid, Spain) and collected in centrifuge tubes. Each sample was injected in quadrupled (n = 4). Matrix-matched calibration curves were prepared by spiking non-contaminated barley flour or C bread extract with OTA standard solution at different concentrations.

### 2.7 Gastric and intestinal extracts analysis and bioaccessibility

For OTA determination, gastric and intestinal extracts were centrifugated at 3849 g for 5 min, filtered by using a 0.22 μm syringe filter and injected into HPLC-FLD. Quantification of OTA was performed by interpolation with matrix-matched calibration curves, prepared by spiking gastric or intestinal control bread digested extracts with OTA standard at different concentrations. OTA bioaccessibility (%) was calculated as the percentage of mycotoxin from the initial bread detected in gastric or intestinal digests. The amount of OTA (μg) in 10 g of bread was calculated from the bread concentration (μg/kg) by conversion factors (× 10/1000) while the amount of OTA (μg) in 100 mL of digest was calculated from digest concentration (μg/L) by conversion factors (× 100/1000).

$$Bioaccessibility=digest\;concentration\;\left(\frac{\mu g}{L}\right)x\;1000÷bread\;concentration\;\left(\frac{\mu g}{L}\right)$$

### 2.8 Cell cultures management

Human colorectal adenocarcinoma Caco-2 cell line was selected for the bioaccessibility study because it represents the most widely used model to simulate the physical and biochemical barrier of the human intestine *in vitro* (De Angelis and Turco, 2011; Ding et al., 2021). Cells were cultured in DMEM with 10% fetal bovine serum (FBS), 1% of 100 U/mL penicillin and 0.1% of 100 mg/mL streptomycin. Incubation conditions were pH 7.4, 5% CO_2_ at 37 °C and 95% air atmosphere at constant humidity. Passages were routinely performed every 2–3 days in 75 cm^2^ plastic flasks with filter screw caps (TPP, Trasadingen, Switzerland) to maintain genetic homogeneity of the cells.

Human T lymphoblastic Jurkat cells are an excellent model for studying immunotoxicity *in vitro*, so they were selected in this work for cytofluorimetry studies (Frangiamone et al., 2023). Jurkat cells were maintained in RPMI 1640 medium supplemented with 1% of 100 U/mL penicillin, 0.1% of 100 mg/mL streptomycin and 10% of FBS. Passages were routinely performed every 2–3 days in 75 cm^2^ plastic flasks in the conditions reported above. For cytofluorimetric studies, 0.5 × 10^6^ Jurkat cells were cultured into 6-well tissue-culture plates and exposed for 7 days to diluted intestinal digests (1/10) in which 0.318 μM was OTA final concentration.

### 2.9 Cell viability assay

MTT assay was used to evaluate differentiated Caco-2 cells viability after the exposure to different concentration of intestinal digests. Caco-2 cells were cultured into 24-well tissue-culture plates: 500 μL of a suspension of 1 × 10^5^ cells/mL was added to each well. Every 3 days the medium was replaced until day 21, which is the differentiation time required for Caco-2 cells to form the *in vitro* gastrointestinal barrier. Five exposure times (24-48-72-96-120 h) were considered using different dilutions of intestinal digests (no diluted; 1/2; 1/4; 1/8; 1/16; 1/32 v/v). For each exposure time, medium containing intestinal digest was removed and a solution of 1 mg/mL MTT was added to each well. After incubation at 37 °C for 4 h, MTT solution was removed and DMSO was added. Absorbance was measured at 620 nm using an BioTek Synergy H1 Multimode Reader (Agilent, VT, USA).

### 2.10 Cytometer setting

All flow cytometry assays were performed using the MACSQuant 16 analyzer (Miltenyi Biotech GmbH, Bergisch Gladbach, Germany) following procedures described in Lázaro et al. (2024). For all flow cytometry experiments (cell cycle, Apoptosis/necrosis, ROS production and mitochondrial mass analysis), the intestinal digests were diluted 1/10 in cell media, wherein the final concentrations were 360 ± 100 nM for OTA digest while 318 ± 140 nM for OTA-GP digest. In all cases, the exposure time for Jurkat cells was 7 days in which cell media was changed every 2–3 days. The software used for flow cytometry assays was Macs quantify version 2.1.

### 2.11 Cell cycle analysis

DNA reagent kit was used for isolating and staining Jurkat cells nuclei. Flow cytometric analysis was used to identify the distribution of cell cycle phases. According to manufactural instruction, Jurkat cells were collected after exposure with intestinal digests and centrifugated. Buffer solution was used to resuspend cellular pellet. During the staining step, cells were incubated for 10 min at room temperature with Solution A (trypsin buffer) and incubated again after the addition of Solution B (trypsin inhibitor and RNase buffer). The last incubation was performed after the addition of Solution C (PI stain solution) at 4 °C for 10 min in dark conditions and analyzed by flow cytometry.

### 2.12 Apoptosis/necrosis analysis

Apoptosis/necrosis outcome was determined using an Annexin V-FITC kit. According to the manufacturer's instruction, Jurkat cells, were collected in a centrifuge tube and Binding Buffer was added to wash cell pellet. Samples were then centrifugated at 300 g for 10 min, cell pellet was resuspended in Binding Buffer containing 5 μL Annexin V-FITC and incubated for 15 min at room temperature in dark condition. Cells were finally washed with binding buffer and Propidium Iodide solution (PI) were added before flow cytometry analysis.

### 2.13 ROS analysis

At the end of exposure time, Jurkat cells were collected in a centrifuge tube and centrifugated at 300 g for 5 min. 5 μM H_2_DCFDA solution was added to resuspend cell pellet and then incubated for 20 min at 37 °C in dark conditions. Samples were then washed twice with PBS and analyzed by flow cytometer. 1 mM tert-Butyl hydroperoxide (TBHP) was used as positive control after 30 min of incubation.

### 2.14 Mitochondrial mass and mitochondrial ROS analysis

Mitochondrial mass was analyzed using MitoTracker green dye. After exposures, 1-3 x 10^5^ Jurkat cells were collected in a centrifuge tube and centrifugated for 5 min at 300 g. Cell pellet was resuspended in staining solution containing 100 nM MitoTracker probe and incubated at 37 °C for 20 min in dark conditions. After staining incubation, samples were centrifugated, resuspended in PBS and analyzed by flow cytometry. Whilst mitochondrial ROS generation in Jurkat cells was monitored using MitoSOX reagent (1 μM as final concentration). 1-3 x 10^5^ Jurkat cells were collected in a 15 ml centrifuge tube and centrifugated. MitoSOX reagent was added to each sample. Samples were washed with PBS and analyzed by flow cytometer. MitoParaquat solution 50 μM was used as positive control after 16 h of incubation.

### 2.15 Statistical analyses of the data

Data were expressed as mean ± SD (n = 3 for bioaccessibility and cell viability evaluations n = 4 for ROS, Apoptosis/Necrosis, and mitochondrial mass analysis, n = 6 for cell cycle analysis). The statistical analysis of the results was performed by Student's t-test for paired samples. p ≤ 0.05 was considered statistically significant. Microsoft Excel® for Microsoft 365 MSO (Version 2306 Build 16.0.16529.20164) was used as statistical software. GraphPad Prism version 9.3.1 for Windows (Boston, Massachusetts USA) was used as graphs builder.

## 3 Results

### 3.1 Contaminated barley flour and bread analysis

The final concentration of OTA in contaminated barley flour used for bread preparation was 149.05 ± 7.89 mg/kg. The concentration of OTA in breads after baking was 15.42 ± 0.95 mg/kg for OTA bread and 15.84 ± 0.58 mg/kg in OTA-GP bread, while no OTA was found in C and GP breads. In gastric digests a concentration of 20 ± 5 nM and 18 ± 8 nM was observed for OTA and OTA-GP digests, respectively. On the other hand, in intestinal digests concentrations of 3.60 ± 0.1 μM and 3.18 ± 0.14 μM were observed for OTA and OTA-GP digests, respectively.

### 3.2 OTA bioaccessibility in gastric and intestinal digests

**Fig. 1:**
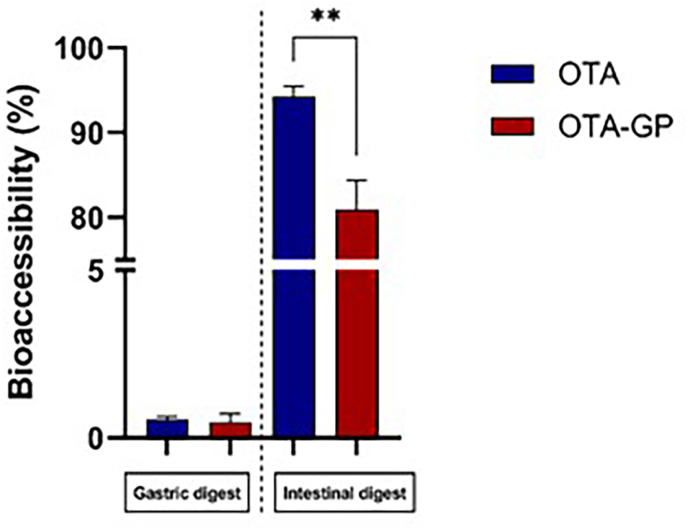
OTA gastric and intestinal bioaccessibility. Data are expressed as mean of three different digestion experiments (n = 3). Significant differences from the control are indicated as p < 0.01 (**).

To evaluate OTA bioaccessibility, an *in vitro* static digestion model was adopted while OTA was quantified by using HPLC-FLD in gastric and intestinal digests. As shown in Fig. 1, OTA bioaccessibility in gastric digests was considerably lower than intestinal digests with no remarkable differences between OTA (0.55 ± 0.09%) and OTA-GP (0.47 ± 0.2%). Conversely, in intestinal digests, a significant difference between OTA and OTA-GP was observed with a reduction of 13% of OTA bioaccessibility in OTA-GP intestinal digest.

### 3.3 Cell viability assay

**Fig. 2:**
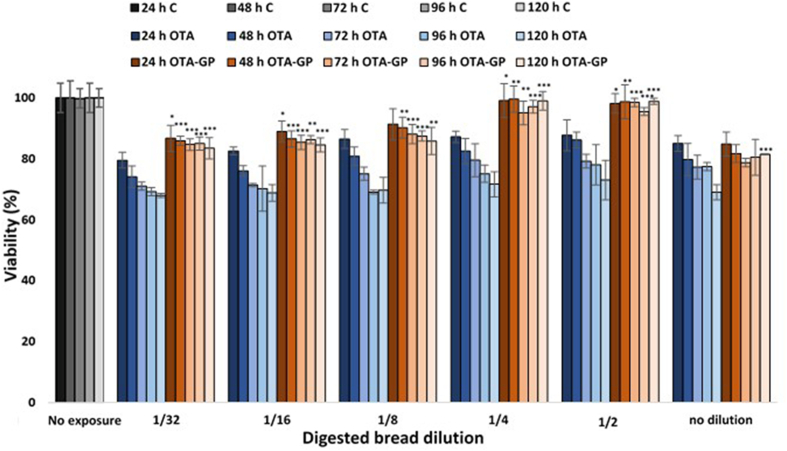
Cell viability in differentiated Caco-2 cells after the exposure to different dilution of intestinal digests for 5 different exposure times. Data graph bars are the mean ± SD (n = 3). Significant differences from OTA and OTA-GP intestinal digest exposure at the same dilution and exposure time are indicated as p < 0.05 (*); p < 0.01 (**); p < 0.001 (***).

OTA toxic effects on Caco-2 cell viability are described in Fig. 2. As expected, OTA reduced cell viability by 20% for each exposure time and dose employed. Indeed, in presence of GP, a significant increase in cell viability in a time- and dose-dependent manner was obtained. Taking exposure time into consideration, it has been observed a cell viability increase of 7–12% after 24 h, 9–17% after 48 h; 13–19% after 72 h; 16–22% after 96 h; 15–27% after 120 h. From the perspective of GP dose, a 7% of cell viability increase was observed in 1/32 diluted intestinal digest, while in 1/2 diluted intestinal digest a 26% increase was obtained.

### 3.4 Cell cycle analysis

**Fig. 3:**
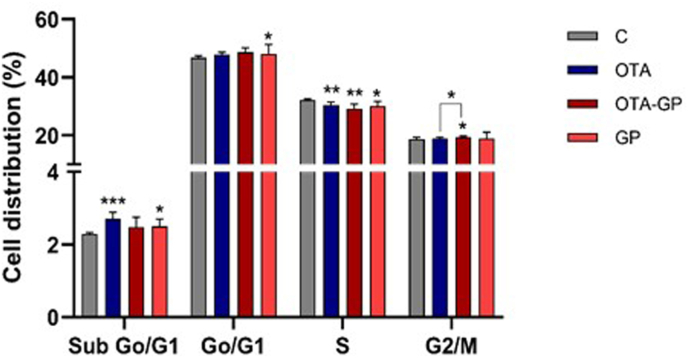
Effect of diluted intestinal digests on Jurkat cells cycle phases (Sub G0/G1; G0/G1; S; G2/M) after 7-days exposure. Exposures using 1/10 intestinal digests in cell media: C, control; OTA, ochratoxin A; OTA-GP, OTA and grape pomace; and GP. Data are expressed as mean ± SD (n = 6) and significant differences from OTA intestinal digest or control are indicated as p < 0.05 (*); p < 0.01 (**); p < 0.001 (***).

The effect of intestinal digests on cell cycle was analyzed by PI staining kit. As shown in Fig. 3, a statistically significant increase in sub-G0/G1 phase was found in cells exposed to OTA digest compared to the control. In contrast, in cells exposed to OTA-GP digest no difference in each cell cycle phase was found in comparison with the control. Significant differences in cell distribution between OTA and OTA-GP were observed in the G2/M phase. Anyways, percentage changes are so small that is unlikely they are translated into biological effects.

### 3.5 Apoptosis/Necrosis pathway analysis

**Fig. 4:**
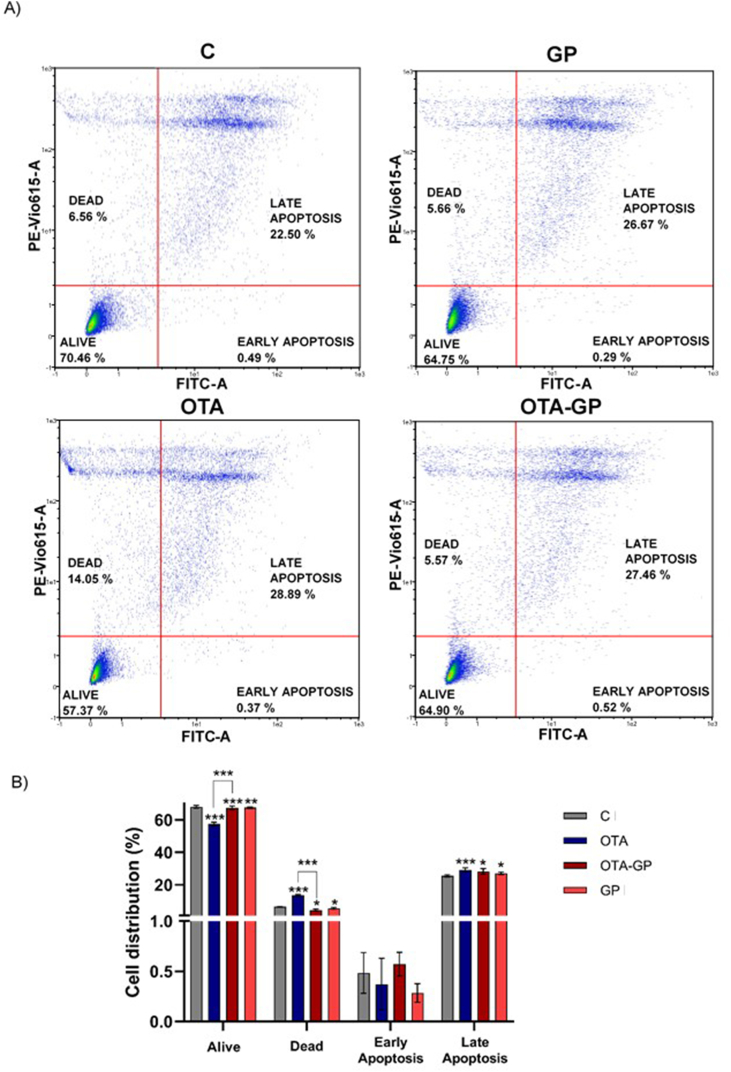
Effect of intestinal digests (0.318 ± 0.014 μM OTA) on apoptosis/necrosis pathway. Exposures for 7 days using 1/10 intestinal digests C, control; OTA, ochratoxin A; OTA-GP, OTA and grape pomace; and GP. (A) Plots from cell apoptosis assay after Jurkat exposure to intestinal digests. (B) Data graph bars are the mean ± SD (n = 4) and significant differences from OTA intestinal digest or control bread are indicated as p < 0.05 (*) and p < 0.001 (***).

The effect of intestinal digests on apoptosis/necrosis outcome is illustrated in Fig. 4. The results show a distribution of 70 ± 2% live cells, 6% dead cells and 22 ± 2% apoptotic cells after exposure to control digest. A significant reduction in the distribution of live cells (57 ± 1%), as well as an increase in dead (14 %) and apoptotic cells (29 ± 1%) was observed for OTA digest compared to the control. In contrast, after exposure to intestinal OTA-GP digest a significant increase in the distribution of live cells (65 ± 1%) and a decrease in dead cells (6%) and in late apoptosis (27 ± 2%) was obtained compared to OTA digest. No significant difference was shown in cell distribution between OTA-GP and GP. A low distribution of cells in early apoptosis was found in all samples (Fig. 4A and B).

### 3.6 ROS analysis

**Fig. 5:**
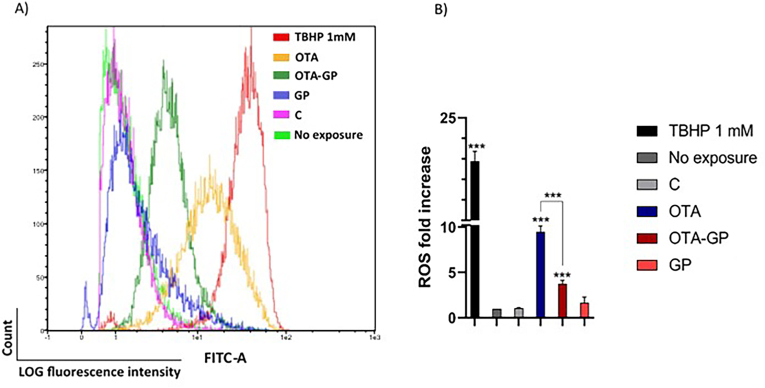
Effect of diluted intestinal digests on radical oxygen species (ROS) generation. Exposures for 7 days using 1/10 intestinal digests C, control; OTA, ochratoxin A; OTA-GP, OTA, and grape pomace; and GP. (A) Representative plots of cell count versus LOG fluorescence of 20.000 events analyzed using flow cytometer for the detection of ROS. (B) The mean fluorescence intensity ± SD (n = 4) expressed as fold increase of the control. Significant differences from OTA intestinal digest or control are indicated as p < 0.001 (***).

Fig. 5 shows the plot graph (A) and histogram (B) related to ROS level identified by flow cytometry expressed as ROS fold increase (FI) compared to the control (unexposed cells). As shown below, exposure to intestinal OTA digest induced 9-fold increase in ROS generation compared to unexposed cells. On the other hand, in OTA-GP digest there was an approximately 3-fold increase in ROS level compared to untreated cells as well as a significant reduction of 37% compared to OTA digest. Cells exposed to the GP intestinal digest (1.26 ± 0.09 FI) and C (1.06 ± 0.20 FI) show no significant differences compared with unexposed cells.

### 3.7 Mitochondrial ROS analysis

**Fig. 6:**
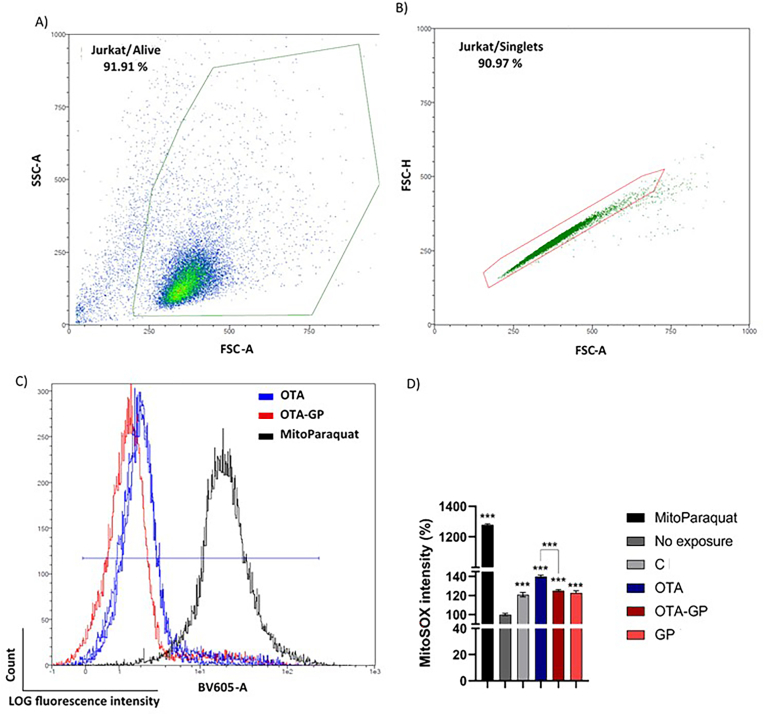
MitoSOX-based flow cytometry detection of mitochondrial reactive oxygen species (ROS) in Jurkat cells after the exposure with diluted intestinal digests. Exposures for 7 days using 1/10 intestinal digests C, control; OTA, ochratoxin A; OTA-GP, OTA, and grape pomace; and GP. Different steps to obtain MitoSOX fluorescence intensity from Jurkat/Singlets: A) Alive Jurkat cells were selected by side scatter (SSC) and forward scatter (FSC) characteristics reported in density plots. B) Singlets Jurkat cells gated by forward scatter characteristics. C) Median fluorescence intensity of Jurkat singlets acquired using B4 channel after exposures. D) Data in the histogram are expressed as mean ± SD (n = 4). Significant differences from OTA intestinal digest or control are indicated as p < 0.001 (***).

To evaluate the effect of intestinal digests on mitochondrial ROS generation, MitoSOX dye was used. Notably, for each intestinal digest, cells show an increase in mitochondrial ROS level compared with unexposed cells (Fig. 6). The greatest increase in ROS production was for OTA digest, with an increase of 40 ± 2%. However, the addition of GP led a clear reduction in mitochondrial ROS production (25 ± 1%), showing its effect also at mitochondrial level.

### 3.8 Mitochondrial mass analysis

**Fig. 7:**
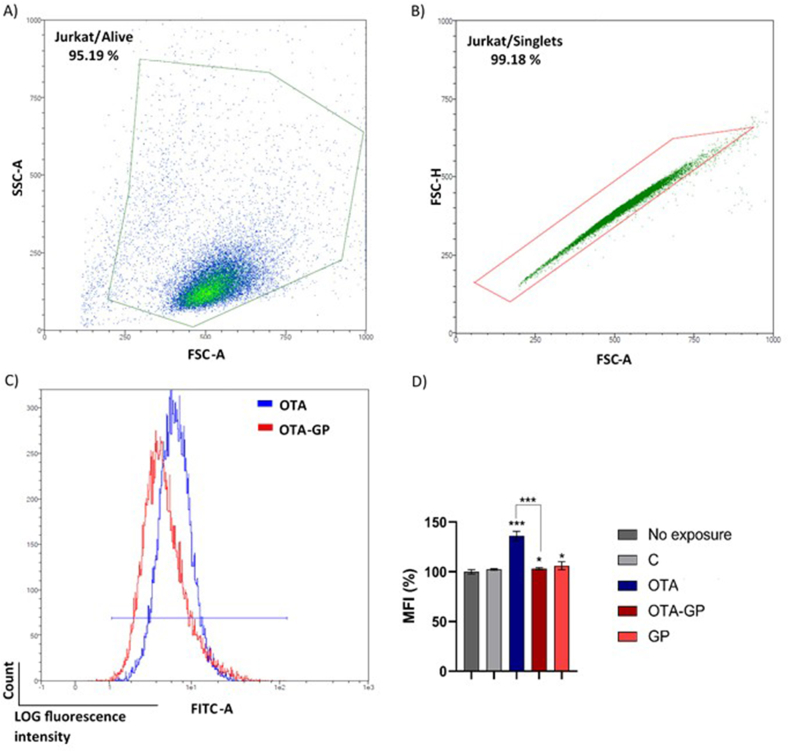
Effect of diluted intestinal digests on mitochondrial mass. Exposures for 7 days using 1/10 intestinal digests C, control; OTA, ochratoxin A; OTA-GP, OTA, and grape pomace; and GP. Different steps to obtain median fluorescence intensity (MFI) from Jurkat/Singlets: A) Selection of alive Jurkat cells by side scatter (SSC) and forward scatter (FSC) characteristics. B) Density plots reports singlets Jurkat cells gated by forward scatter characteristics. C) Median fluorescence of Jurkat singlets acquired using FITC channel. D) MFI ± SD (n = 4) of MitoTracker dye measured after incubation of Jurkat cells with intestinal digests. Significant differences from OTA intestinal digest or control are indicated as p < 0.05 (*) and p < 0.001 (***).

Mitochondrial mass analysis was performed by incubation of Jurkat cells, exposed to intestinal digests, with MitoTracker dye. In Fig. 7 the effect of diluted intestinal digests on mitochondrial mass is shown. A significant increase of 36% in mitochondrial mass was found after cell exposure to OTA digest. Interestingly, an increase of only 3% in mitochondrial mass was found upon exposure of cells to OTA-GP, with a significant reduction of 24% when compared with OTA.

## 4 Discussion

The main goal of this study was to investigate the possible GP use as a novel biosorbent material and source of polyphenols in cereal-based products with the purpose to mitigate OTA bioaccessibility and its toxic effects. The experimental model were human gastrointestinal and lymphocytic cell lines. To date, it has been reported that OTA is present in wine and can interact with GP (Gambacorta et al., 2016). Solfrizzo et al. (2010) also proposed a procedure involving the use of GP to decrease OTA levels in must and wine. The procedure consisted in repassing the contaminated musts or wines on the pomace, which led to a 65% OTA reduction. These outcomes supported the high GP affinity for OTA. Accordingly, in the present work, freeze-dried Cabernet GP was used as putative functional ingredient against OTA considering its total phenolic content of 2064.64 mg/g with a major contribution from p-coumaric acid and gallic acid (Mangiapelo et al., 2024).

Firstly, the possible beneficial GP role in reducing OTA-bioaccessibility was assessed. Moving to results, the low OTA concentration in gastric compartment indicated that OTA release from bread occurs mainly in the intestine owing to its physicochemical properties and a less acidic pH level than the stomach (Escrivá et al., 2021). In the intestinal phase, it has been observed a bioaccessibility value of 94% for OTA, which is strictly like other studies that used cereals, baby foods and cornmeal (in all cases, values above the 85%) (Rebellato et al., 2021; Versantvoort et al., 2005; Nobre et al., 2022). On the other hand, an intestinal value of 81% was obtained for OTA-GP digest, showing a significant beneficial GP effect on OTA-bioaccessibilty. This phenomenon can be associated with the chemical composition of GP, rich in phenolic compounds, that generates polar non-covalent interactions with OTA in the gut, such as electrostatic interactions and hydrogen bonds, by limiting its absorption (pH 6.5 ± 0.2) (Avantaggiato et al., 2014).

Regarding cell viability, Caco-2 cells were differentiated to reproduce a gastrointestinal *in vitro* barrier and therefore a more realistic scenario. In accordance with our findings, several studies confirmed how short exposures to high doses of OTA (24–48 h; 5–100 μM) on undifferentiated and differentiated Caco-2 decreased cell viability in a concentration and time dependent manner (Cano-Sancho et al., 2015; Gonzalez-Arias et al., 2017; Gao et al., 2016; Guerra et al., 2005). In this study, the cytoprotective *in vitro* role of GP was also evaluated. Indeed, the addition of this functional ingredient in contaminated bread promoted a significant increase of cell viability, showing its protective effect against OTA-cytotoxicity. Similar results were found by Frangiamone et al. (2022b), exposing differentiated SH-SY5Y cells to intestinal digests with low doses of OTA and functional ingredients such as pumpkin and fermented whey. Authors observed how the addition of bioactive compounds in contaminated digests mitigated OTA toxicity increasing cell viability by 20%. However, it was the first time, in which *in vitro* GP cytoprotection was tested, but these results indicated the clear GP ability in limiting OTA-induced cell mortality.

Moving to blood cell line, it should be noted that OTA can also induce cell death through cell cycle disruption. However, in the present study, no clear differences were observed between control and OTA-digest for any phase of Jurkat cell's cycle (Fig. 3). The likely concentration of OTA in the intestinal digest may not be sufficient to induce changes in Jurkat cell cycle, suggesting another mechanism of action for its toxic effects. Therefore, it is not possible to compare these results with previous ones because it was typical in toxicology to study doses 10 times higher than the one obtained in this study following a simulated digestion protocol (Asci Celik et al., 2020; Zhang et al., 2020).

OTA exposure had an inhibitory effect on Jurkat T cell proliferation with a strong increase in the number of apoptotic and necrotic cells (Skrzydlewski et al., 2022). Additionally, Xu et al. (2017) showed a 40% increase of late apoptotic macrophages after OTA exposure (0.5–1 μM) for 24 h whereas Gan et al. (2022) obtained similar results by exposing the same cell line to high OTA concentrations (5–7 μM) for 48 h. In line with these outcomes, although a longer exposure time and lower doses were used, this study demonstrated how the percentage of necrotic and late apoptotic cells reaches its peak after OTA exposure (Fig. 4a and b), indicating that cell death can be considered a putative mode of action (MoA) whereby OTA promoted toxicity in human lymphoblastic cell line. Whilst, the administration of GP, similarly to other functional ingredients used against this toxin such as *Allium sativum* and curcumin, clearly reduced the apoptotic and necrotic cells ratio promoted by OTA (5 % and 10%, respectively), demonstrating again its antiapoptotic effect against OTA induced cell death not only in Caco-2 but also in Jurkat T cells (Damiano et al., 2021; Lázaro et al., 2024).

Furthermore, it has been reported that one of the main MoA by which OTA induced toxicity *in vitro* and *in vivo* is the promotion of oxidative stress with ROS generation (Wen et al., 2016; Sun et al., 2023). In human blood mononuclear cells, exposure to OTA (5–20 μM; 24h) promoted ROS production, decreased antioxidant activity of glutathione, and increased 8-hydroxydeoxyguanosine level, that is the main biomarker of DNA oxidative stress (Liu et al., 2012). Likewise, low OTA doses (0.5 μM; 24h) fostered immune toxicity in porcine macrophages by ROS relative TLR4/MyD88 signaling pathway (Xu et al., 2017). In chicken heterophils, oxidative stress generation was related to NADPH oxidase, ERK, and p38 MAPK signaling pathways activation. Authors also affirmed that 2h exposure to OTA (5–20 μM) is sufficient to trigger an aberrant immune response that, in case of prolonged toxin exposure, can lead to an hyporeactivity of the immune system against pathogen infection (Han et al., 2019). By using the same cell line and similar exposure time and toxin doses (100 nM; 7 days), Frangiamone et al. (2023) showed at transcriptional level how the combined exposure to OTA-AFB1 triggered a type of programmed necrosis, named ferroptosis, with lipid peroxidation and inhibition of glutathione 4 peroxidase activity. It is also known that ROS are mainly produced in mitochondria during oxidative respiration by electron transport chain (ETC) (Zhao et al., 2019). Indeed, Wang et al. (2017) obtained a strong increase of both mitochondrial ROS production and permeability with cytochrome *c* release and apoptosis in human intestinal epithelium after 12 h exposure to OTA (2–8 μM). Similar findings were obtained by Gan et al. (2016) exposing PK15 cells to OTA (100 nM; 60 h). Herein, mycotoxin promoted a significant increase of ROS generation at mitochondrial level by Nrf2 signaling inhibition with alteration of antioxidants enzymes activity. In these studies, authors correlated the increase of mitochondrial ROS with ETC loss.

In this context, it should be noted that ROS generation can lead to mitochondrial mass increase (Yu et al., 2006). Indeed, a short exposure to OTA in retinal mice cells for 3 days not only caused apoptosis, oxidative stress, and mitochondrial dysfunction but also a change in mitochondria morphology, which shifted from tubular to spherical one, typically associated with human diseases (Fu et al., 2024). Likewise, in GES-1 human gastric cells, 24 h OTA-exposure (10 μM) impaired mitochondrial function and permeability, by promoting ROS generation, apoptotic and autophagic cell death. In addition, toxin significantly increased mitochondrial mass and volume, which may be inversely correlated with mitophagy but directly related to cell survival and malignant transformation in GES-1 cell line (Li et al., 2019).

In accordance with these findings, in the present study, OTA exposure significantly increased ROS generation, mitochondrial ROS and mass (Fig. 5, Fig. 6, Fig. 7). Therefore, oxidative stress via mitochondria dysfunction such as morphological changes and possible ETC leakage may be considered a possible MoA by which OTA promoted immune toxicity *in vitro.* However, GP supplementation showed a strong ability in reduced ROS production, mitochondrial ROS and mass, demonstrating its clear antioxidant effect against OTA-toxicity *in vitro* (Fig. 5, Fig. 6, Fig. 7). Whilst GP beneficial role is associated with the high content of phenolics compounds, the specific GP MoA can be correlated to its activity as free radical inhibitor as well as with the scavenging ability on superoxide anion, hydroxyl radical and hydrogen peroxide. Additionally, GP can play an inhibitor effect on iron induce lipid peroxidation while mitochondria can be considered a possible target of GP-MoA (Untea et al., 2023). Indeed, 30 days grape waste administration has been reported to reduce AFB1 induced mitochondrial dysfunction, inflammation, and oxidative stress by improving antioxidants enzymes activity in pig spleen and liver. Authors concluded that grape waste can be used by food industry as novel functional ingredient to counteract the worldwide issue of mycotoxins contaminations (Taranu et al., 2019; Marin et al., 2020). Overall, these results underline the strong *in vitro* antioxidant and antiapoptotic effect of GP addition in OTA-contaminated bread as well as its potential use as biosorbent material in food to limit mycotoxin contamination and therefore limit human health risk from fungal toxicity.

## 5 Conclusion

In this study, the effect of Cabernet GP on reducing OTA bioaccessibility was investigated by using digested bread extracts, alongside GP potential preventive role against OTA in human Caco-2 and Jurkat T cells. As regard *in vitro* OTA effects, it has been showed that cell death, ROS generation and mitochondrial dysfunction can be assumed to be putative MoA whereby OTA-induced toxicity. Conversely, a significant reduction in OTA bioaccessibility was observed upon GP inclusion in bread formulation. GP addition also demonstrated a protective beneficial role against OTA-induced cytotoxicity in human cells by mitigating cell death and ROS production. In addition, GP limited OTA-induced mitochondrial oxidative stress and mass changes which in turn can indicate as mitochondria may be a potential target of GP-MoA. However, no remarkable changes were found in cell cycle, suggesting as this biological process might not be implicated in GP-MoA. Overall, these findings suggest the strong antiapoptotic and antioxidant effect of Cabernet GP as well as its possible application as functional ingredient in food industry to limit OTA-contamination and toxicity. Further research is also needed to better explore GP-MoA and its putative *in vivo* use, but results are promising.

## CRediT authorship contribution statement

**Luciano Mangiapelo:** Investigation, Data curation, Writing – original draft. **Massimo Frangiamone:** Investigation, Formal analysis. **Pilar Vila-Donat:** Data curation, Methodology, Writing – review & editing. **Denisia Paşca:** Formal analysis, Visualization. **Federica Ianni:** Validation, Visualization. **Lina Cossignani:** Writing – review & editing. **Lara Manyes:** Conceptualization, Project administration, Supervision.

## Declaration of competing interest

The authors declare that they have no known competing financial interests or personal relationships that could have appeared to influence the work reported in this paper.

## Data Availability

Data will be made available on request.
